# Mangafodipir Protects against Hepatic Ischemia-Reperfusion Injury in Mice

**DOI:** 10.1371/journal.pone.0027005

**Published:** 2011-11-02

**Authors:** Romain Coriat, Mahaut Leconte, Niloufar Kavian, Sassia Bedda, Carole Nicco, Christiane Chereau, Claire Goulvestre, Bernard Weill, Alexis Laurent, Frédéric Batteux

**Affiliations:** 1 Laboratoire d'immunologie, EA1833 Université Paris Descartes, Sorbonne Paris Cité, Faculté de Médecine, AP-HP, Hôpital Cochin, Paris, France; 2 Service d'hépatogastroentérologie, Université Paris Descartes,Sorbonne Paris Cité, Faculté de Médecine, AP-HP, Hôpital Cochin, Paris, France; 3 Service de Chirurgie Digestive, Université Paris Descartes, Sorbonne Paris Cité, Faculté de Médecine, AP-HP, Hôpital Cochin, Paris, France; 4 Laboratoire d'immunologie biologique Université Paris Descartes, Sorbonne Paris Cité, Faculté de Médecine, AP-HP, Hôpital Cochin, Paris, France; 5 Service de Chirurgie Digestive, Hôpital Henri Mondor, Université Paris XII, Créteil, France; University of Colorado Denver, United States of America

## Abstract

**Introduction and Aim:**

Mangafodipir is a contrast agent used in magnetic resonance imaging that concentrates in the liver and displays pleiotropic antioxidant properties. Since reactive oxygen species are involved in ischemia-reperfusion damages, we hypothesized that the use of mangafodipir could prevent liver lesions in a mouse model of hepatic ischemia reperfusion injury. Mangafodipir (MnDPDP) was compared to ischemic preconditioning and intermittent inflow occlusion for the prevention of hepatic ischemia-reperfusion injury in the mouse.

**Methods:**

Mice were subjected to 70% hepatic ischemia (continuous ischemia) for 90 min. Thirty minutes before the ischemic period, either mangafodipir (10 mg/kg) or saline was injected intraperitoneally. Those experimental groups were compared with one group of mice preconditioned by 10 minutes' ischemia followed by 15 minutes' reperfusion, and one group with intermittent inflow occlusion. Hepatic ischemia-reperfusion injury was evaluated by measurement of serum levels of aspartate aminotransferase (ASAT) activity, histologic analysis of the livers, and determination of hepatocyte apoptosis (cytochrome c release, caspase 3 activity). The effect of mangafodipir on the survival rate of mice was studied in a model of total hepatic ischemia.

**Results:**

Mangafodipir prevented experimental hepatic ischemia-reperfusion injuries in the mouse as indicated by a reduction in serum ASAT activity (*P*<0.01), in liver tissue damages, in markers of apoptosis (*P*<0.01), and by higher rates of survival in treated than in untreated animals (P<0.001). The level of protection by mangafodipir was similar to that observed following intermittent inflow occlusion and higher than after ischemic preconditioning.

**Conclusions:**

Mangafodipir is a potential new preventive treatment for hepatic ischemia-reperfusion injury.

## Introduction

### Ethics Statement

Animals received human care in compliance with institutional guidelines under the permit number 75–1302 delivered to Dr Carole Nicco, PhD, the 04/06/2007.

Blood loss and transfusions during liver resection have a deleterious impact on both short and long-term outcomes [1], [2]. To minimize intra-operative bleeding, surgical management of hepatectomy requires pedicular clamping [3]. The common drawback of clamping is hepatic ischemia-reperfusion (I/R) injury, especially when the liver is affected by chronic hepatitis or cirrhosis, with a risk of poor postoperative outcome [4]. Two surgical strategies have been developed to minimize I/R injuries: intermittent clamping (IC) and ischemic preconditioning (IP).

IC consists of an intermittent inflow occlusion followed by short periods of reperfusion and has demonstrated a protective effect [5]–[7]. A drawback inherent to IC is blood loss during each period of reperfusion and the increased operative time [8]. The alternative to IC is IP that consists of a brief period of ischemic reperfusion applied prior to the prolonged ischemic insult. IP has demonstrated a protective effect during liver resection in humans [9]. Studies in animal models of ischemia reperfusion have suggested that the protective effect of IP is mediated by an enhancement in endogeneous anti-oxidative stress mechanisms [10], [11].

Liver injury following I/R has a biphasic pattern [12]. In the initial phase, 0.5–2 h after the onset of reperfusion, reactive oxygen species (ROS) are released and Kupffer cells and hepatocytes are activated. The late phase corresponds to self-aggravating inflammatory injury inducing ROS [12]. Hepatocytes injuries are most likely initiated by ROS and extracellular chemokines [13]. ROS have been shown to exert a central role in contributing to tissue injury after reperfusion of the ischemic liver [14]–[16].

Superoxide anions (O_2_°^−^) originating from either the mitochondrial respiratory chain or various cytosolic enzymes, such as xanthine oxidase or NADPH oxidase, are generated by ischemic hepatocytes. Superoxide anions are detoxified by superoxide dismutase (SOD) that convert O_2_°^−^ into H_2_O_2_ which is then detoxified by catalase, glutathione peroxydase, or thioredoxine [17], [18]. The overproduction of ROS leads to lipid peroxidation, damages of mitochondrial membrane [19], release of cytochrome c into the cytoplasm followed by caspase-3 activation, and finally, to hepatocyte apoptosis [20]. Endogenous antioxidant compounds, such as superoxide dismutase, catalase, and glutathione can limit the effects of ROS but quickly become overwhelmed by the large amounts of ROS produced.

Because the oxidative stress plays such an important role in I/R injury, we hypothesized that the administration of a molecule endowed with antioxidative properties could be a valuable treatment of I/R injury. SOD, catalase and GSH reductase have been administered in animal models to counterbalance the endogenous enzymatic depletion during hepatic ischemia-reperfusion [21], [22]. Although the targeted delivery of SOD and catalase to Kuppfer cells after mannosylation or succinylation could prevent hepatic injury [18], the effectiveness of an exogenous enzymatic supply was controversial probably because of the insufficient delivery to the target sites. Therefore, we chose to test mangafodipir, a non peptidic enzymatic mimic with a high level of liver intracellular penetration. Mangafodipir (MnDPDP), a contrast agent used in magnetic resonance imaging of the liver, comprises a fodipir moiety, a chelate of manganese resulting from the condensation of two pyridoxal 5′ phosphate molecules [23]. The fodipir moiety binds to the pyridoxal 5′ phosphate receptor on hepatocytes and ensures a high intrahepatic concentration of mangafodipir in the liver. In addition to the known capability of fodipir to increase GSH levels under a variety of oxidative conditions [24]–[26], we have shown that mangafodipir displays pleiotropic antioxidant properties. Indeed, this molecule is endowed with SOD-, catalase-, and glutathione reductase-like activities that allow both detoxification of mitochondrial ROS and regeneration of the GSH pool [27]. Those properties explain the effectiveness of mangafodipir in the treatment of APAP-induced acute liver failure in mice [28]. The aim of our study was, therefore, to identify the therapeutic activity of mangafodipir in a mouse model of hepatic I/R injury, versus IC and IP.

## Methods

### Chemicals

All chemicals were from Sigma (Saint Quentin Fallavier, France) except for mangafodipir (MnDPDP, Teslascan®, Amersham Health, Amersham, UK).

### Animals

BALB/c female mice between 6 and 8 weeks of age were used in all experimental groups (Iffa Credo, L'Arbresles, France). Animals received human care in compliance with institutional guidelines under the permit number 75–1302 delivered to Dr Carole Nicco, PhD, the 04/06/2007. Hepatic ischemia was performed as follow: animals were anesthetized with an intraperitoneal injection of Avertine (10 mg/kg) and laparotomy was performed. Ischemia of the left lateral and left median lobes was induced by a microvascular clamp for 90 minutes. Reperfusion was initiated by removing the clamp [29].

### Experimental Design

The following groups of mice were studied: Group 1: sham (anesthesia and laparotomy) (n = 9); group 2: control (90 minutes of ischemia) (n = 9); group 3: (as group 2 with intraperitoneal administration of 10 mg/kg of mangafodipir 30 minutes prior to ischemia) (n = 9); group 4: IP(as group 2 but with preconditioning induced by 10 minutes of ischemia followed by 15 minutes of reperfusion [30]) (n = 9); group 5: IC (six cycles of 15 minutes of ischemia followed by 5 minutes of reperfusion [30]) (n = 9).

Mice were injected intraperitoneally with 10 mg/kg of mangafodipir, which corresponds to the concentration used in humans as a contrast agent [31].

Twenty four hours following ischemia, the animals were sacrificed, blood was collected for measurement of serum transaminase activity, and livers were removed from the peritoneal cavities for histopathological and biochemical studies.

### Mitochondrial and Cytosolic Production of O2°−

The effects of mangafodipir on intracellular ROS production were assessed *in vitro* in human (HepG2) hepatocellular carcinoma cell line. Cytosol and mitochondria from HepG2 cells (15 x10e6 cells) were seeded in 24-well plates (Costar, Corning, Inc., Corning, NY) and incubated for 18 hours with mangafodipir or with culture medium alone. Levels of intracellular superoxide anion O2°- were assessed spectrofluorometrically (Packard Bioscience, Boston, MA, USA) by oxidation of dihydroethidium (DHE) (Molecular Probes, Leiden, The Netherlands) as previously described [32]. The levels of O2°- were calculated in each sample as follows: ROS rate (arbitrary units/2*10e6 cells)  =  (fluorescence intensity [arbitrary units].

### Serum Aminotransferase activity

Serum activity of aspartate aminotransferase (ASAT) was used as a marker of hepatocyte cytolysis. ASAT activity was quantified using a standard clinical automatic analyser (Hitachi type 747, Roche Diagnostics, Meylan, France).

### Histological examination of livers

Livers were fixed overnight at 4°C in 4% paraformaldehyde in PBS, dehydrated, paraffin-embedded, and cut at 4-µm thickness. Hematoxylin-eosin-stained sections were used for histopathological evaluation of hepatic injuries. Two pathologists who were not aware of which animals received which treatment examined slides.

### Lipid peroxidation assay

The concentrations of 4-hydroxyalkenals (4-HNE) and malondialdehyde (MDA) were measured in whole liver homogenates using the lipid peroxidation kit from Calbiochem (Calbiochem, Paris, France): whole liver homogenates were mixed with methanol: acetonitrile and N-methyl-2-phenylindole to yield a purple chromophore, which was measured spectrophotometrically at 586 nm. The level of lipid peroxydation was expressed as the amounts of 4-HNE + MDA per mg of proteins.

### Measurement of hepatic GSH content

GSH levels in liver tissue were measured by the method of Baker et al [33]. Briefly, 50 *µ*l of whole liver extract (1 mg/mL) or of reduced GSH (Sigma) as standard were added to 100 *µ*L of a reaction mixture containing 5 mL of 1 mM DNTB, 5 mL of 1 mM NADPH, 5.75 mL of 100 mM NaPO_4_, 1 mM EDTA buffer, pH 7.5 and 0.1 mL of GSH reductase (200 U/mL). GSH levels were determined by measuring absorbances at 405 nm.

### Cytochrome c determination

Cytochrome c concentration was determined on cytosolic fractions or enriched mitochondrial fractions from livers of mice. The amount of proteins in each lysate was measured using the BSA microbiuret assay (Pierce, Bezons, France). The rat/mouse Cytochrome c ELISA from R&D (Abingdon, Oxon, UK) was used to determine the levels of cytochrome c in each fraction. Results were expressed as pg of cytochrome c per µg of proteins.

### Measurement of caspase-3 activity

Caspase-3 activity was measured on cytosolic fractions from livers of mice using 400 µM of chromogenic substrate Ac-DEVD-pNA (Calbiochem) and 200 µM of cytosolic fraction. Caspase-3 activity was calculated as the mean of the duplicate test wells minus the value obtained for the control wells containing 200 µM specific inhibitor.

### Animal Survival

The rate of mouse survival was evaluated in groups 2 and 3 after total hepatic ischemia (22). Segmental hepatic ischemia (70%) was followed by the resection of the non ischemic liver lobes (30%). For the survival time courses, animals were observed for 30 days and sacrificed when they appeared moribund. The moribund state has been appreciated in a blinded manner by two observers who were not aware of which animals had received which treatment.

### Statistical analysis

Results were expressed as means +/− standard error (SEM). The statistical significance of differences in the groups was analysed by chi-square tests for incidence data. Paired Student's *t*-test was used for comparison of means between two groups. A level of *P*<0.05 was accepted as significant. * *p*<0.05; ** *p*<0.02; *** *p*<0.01; **** *p*<0.001 *vs* untreated ninety minutes' ischemia controls.

## Results

### Serum enzymatic activities

**Figure 1:**
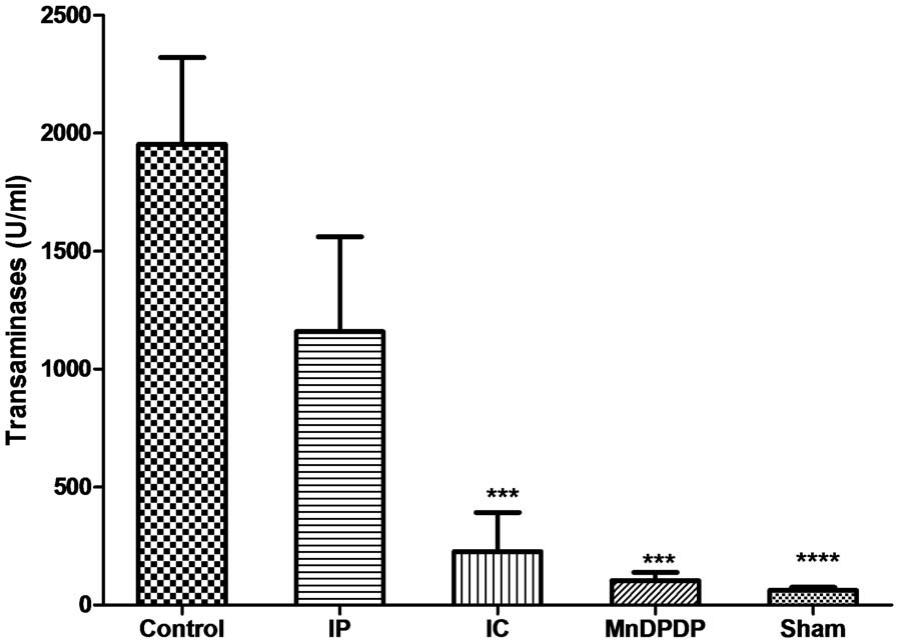
Mangafodipir and intermittent clamping prevent the increase in transaminase activities following reperfusion injury. Serum levels of aspartate amino-transferase activities (ASAT) served as markers of hepatocyte injury. ASAT levels were measured after 24 hours of reperfusion. Bars represent means ± SEM, nine mice in each group.

Ninety minutes' ischemia (control) induced a 40-fold increase in serum transaminase activityies compared to the sham-operated group. Compared to the untreated ninety minutes' ischemia control group, ASAT activities were significantly lower after administration of mangafodipir and IC (*P*<0.01 and p = 0.01, respectively), and not significantly different from the basal level observed in the sham-operated group. ASAT activities in the IP group were lower than in the control group, but the difference between the two groups did not reach significance (Fig. 1).

### Liver histological examination

**Figure 2:**
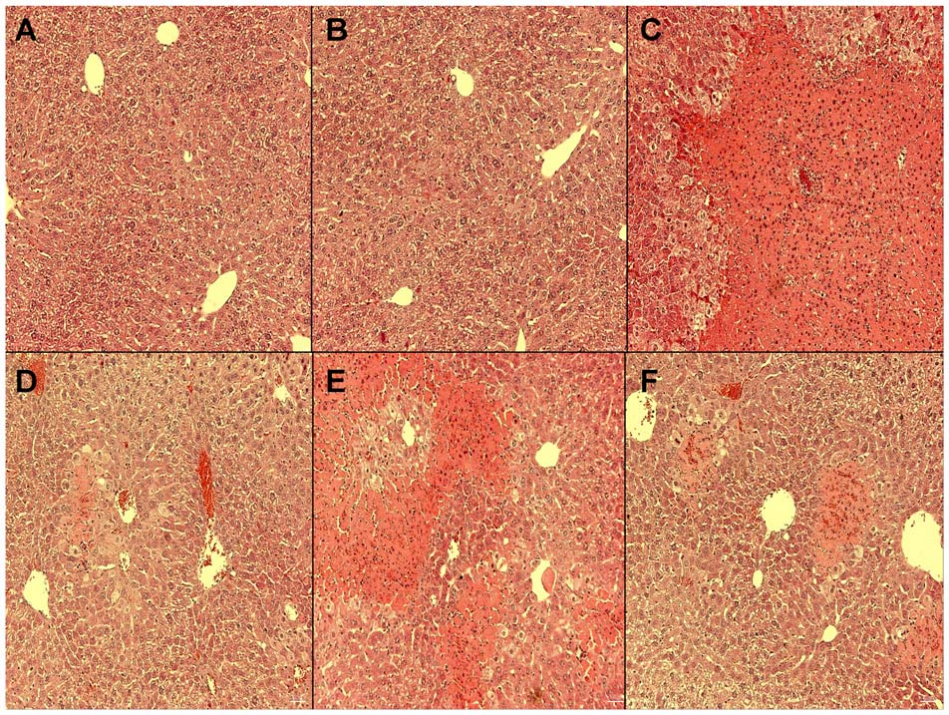
Mangafodipir and intermittent clamping prevent histological lesions of the liver following reperfusion injury. Hematoxylin-eosin-stained sections were used for histopathological evaluation of hepatic injuries. The sections were examined at 400-fold magnification. Representative livers from normal BALB/c mice (A), Sham-operated mice (B), ninety minutes' ischemia control group (C), Mangafodipir (MnDPDP)-treated group (D), IP-treated group (E) and IC-treated group (F).

After 90 minutes of continuous ischemia, large confluent areas of tissue lysis with blood congestion in the sinusoids and leukocyte infiltrates were observed. In the IP liver, limited and focal areas of hepatocyte necrosis were also observed (Fig. 2), whereas the parenchyma was almost normal after mangafodipir or IC treatment.

### Lipid Peroxidation assay

**Figure 3:**
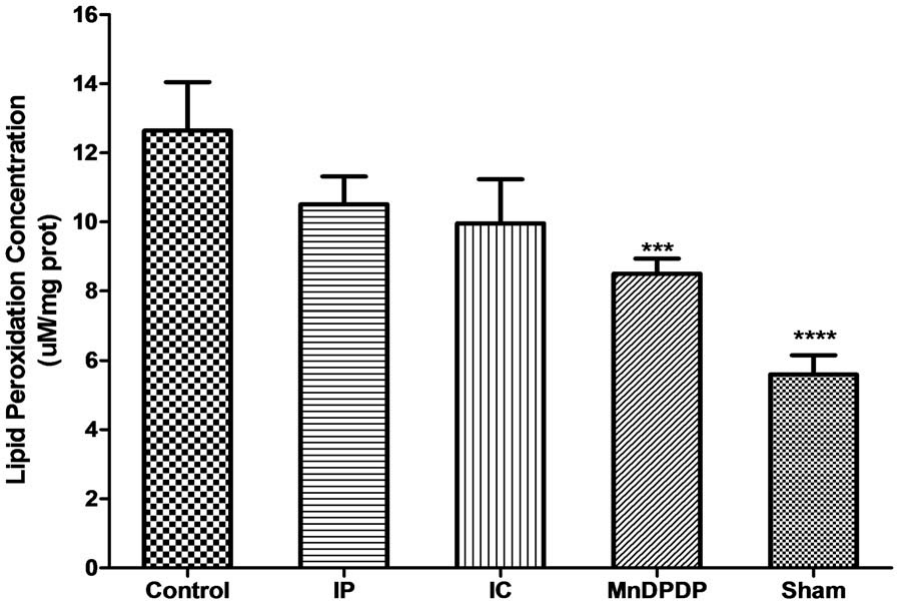
Prevention of lipid peroxidation following ischemia-reperfusion of the liver using on mangafodipir and intermittent clamping. The concentrations of 4-hydroxyalkenals (4-HNE) and malondialdehyde (MDA) were measured spectrophotometrically in whole liver homogenates. The level of lipid peroxidation was expressed as the amounts of 4-HNE + MDA per mg of proteins. Bars represent means ± SEM; nine mice in each group.

Lipid peroxidation has been used as an indirect measurement of oxidative damage induced by ROS (24). Levels of 4-HNE and MDA were significantly higher in the ninety minutes' ischemia control group than in the sham-operated group (13.3±1.7 µM/mg *versus* 5.4±0.5 µM/mg, *P*<0.001) after liver reperfusion. In animals treated by IC or IP, lipoperoxidation was not significantly reduced (9.8±1.5 µM/mg with IC and 10.2±1.1 µM/mg with IP. Only mangafodipir significantly reduced lipoperoxidation *versus* the ischemia control group (7.6±0.3 µM/mg, *P*<0.01) (Fig 3).

### Measurement of hepatic GSH content

**Figure 4:**
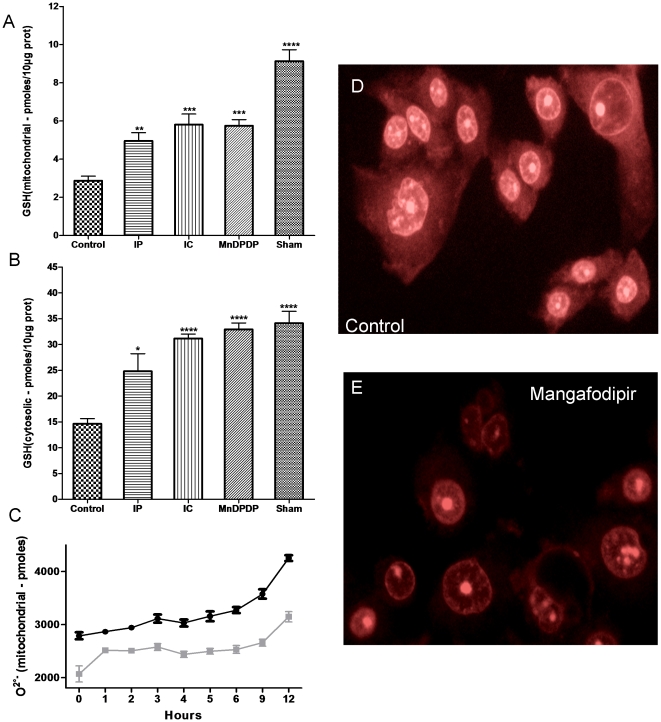
Prevention of glutathione depletion in the mitochondria (A) and in the cytoplasm (B) of hepatocytes following reperfusion injury. The concentrations of glutathione in cytoplasm and mitochondria of mouse liver were measured spectrophotometrically in whole liver homogenates. The levels of reduced gluthatione were expressed as pmol per 10 µg of proteins. Bars represent means ± SEM, nine mice in each group. The O2°- depletion in the mitochondria (C) of HepG2 cells induced by mangafodipir (400 µM) was assessed in a kinetic experiment. Immunofluorescence microscopy of HepG2 cells stained with oxidation of dihydroethidium (DHE) and treated for 18 h or not with mangafodipir. (D–E) A decreased intensity of cytosol with mangafodipir in mangafodipir treated cells was observed.

In the control group with continuous inflow occlusion, the cytosolic GSH content was decreased by 55% versus the sham-operated group. Treatment with mangafodipir, IC or IP, decreased mitochondrial GSH content by only 4%, 14% and 30%, respectively (P<0.001, 0.001 and 0.05, respectively *versus* the ninety minutes' ischemia control group). The GSH content in the mitochondria of liver cells was also modified following continuous inflow occlusion and was significantly lower than in the sham-operated group (P<0.001). Mangafodipir, IC and IP increased the mitochondrial GSH content *versus* the ninety minutes' ischemia control group (P<0.01, 0.001 and 0.02, respectively) (Fig 4A and 4B).

### Cellular Mediators of Apoptosis

To further evaluate that mangafodipir inhibit cysolic and mitochondrial ROS, levels of intracellular superoxide anion O2°- in mitochondria and cytosol from HepG2 cells were assessed and confirm a 29% and 28% ROS level decreases in mitochondria and cytosol, respectively. (figure 4C–D)

### Cellular Mediators of Apoptosis

**Figure 5:**
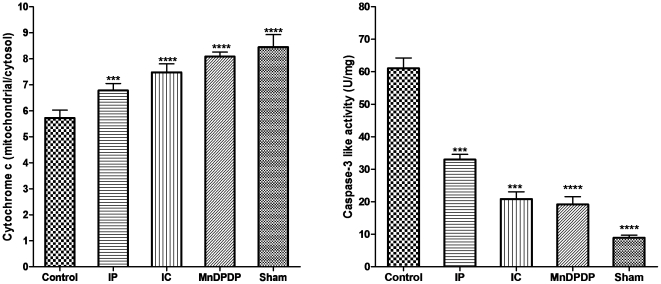
Prevention of hepatocytes apoptosis following reperfusion injury. The mitochondrial/cytosol cytochrome c ratio (A) and caspase-3 activities were measured in the livers of the various experimental groups and controls. Caspase-3 like activities were expressed as Units per mg of proteins. Bars represent means ± SEM, nine mice in each group.

To further evaluate the mechanisms leading to liver cell injuries, we evaluated apoptotic liver cell death by measuring the cytochrome c released from mitochondria into the cytosol, and by assaying cytosolic caspase-3 activity. Ischemia/reperfusion injuries were associated with mitochondrial collapse, as revealed by the significant 35% decrease in the mitochondrial/cytosolic cytochrome c ratio in the ninety minutes' ischemia control group *versus* the sham-operated group (P<0.001).The decrease in the mitochondrial/cytosolic ratio was reduced by IP (24%), IC (16%) and mangafodipir (9%) *versus* the ninety minutes' ischemia control group (P<0.001, 0.001 and 0.01, respectively) (Fig 5A). The mitochondrial collapse following reperfusion injury is followed by an increase in caspase-3 activation, as exemplified by the 9-fold increase in caspase-3 activity in the ischemia control group *versus* the sham-operated group (P<0.001). Mangafodipir, IC and IP increased by 2.3-, 3.2- and 4.8-fold the caspase-3 activity (P<0.001, 0.01 and 0.01, respectively) *versus* the ischemia control group (Fig 5B).

### Effect of mangafodipir on the Survival Rate of Mice

In the ischemia control group, 8 mice of 9 survived no longer than 7 days and only one mouse survived longer than 30 days (Table 1). By contrast, all animals survived more than 30 days in the group treated by mangafodipir (P<0.001).

**Table 1:** Increasing the survival rate of mice by mangafodipir following reperfusion injury of the liver.

90 minutes of ischemia	7 days overall survival	30 days overall survival
Continuous inflow occlusion (control)	11%	0%
Mangafodipir therapy prior to ischemia	100%	100%

* Animal survival was evaluated using the model of total hepatic ischemia (37).

The rate of mouse survival was evaluated in control group (90 minutes of ischemia; n = 9) and in control group with intraperitoneal administration of 10 mg/kg of mangafodipir 30 minutes prior to ischemia (n = 9) [40]. For the survival time courses, animals were sacrificed when they appeared moribund.

## Discussion

The damage caused to the liver by I/R is a limiting factor in many clinical settings such as liver surgery, transplantation, and low-flow states. Because ROS and oxidative stress have been shown to play a major role in organ I/R injury, the purpose of this study was to test the hypothesis that mangafodipir, an antioxidant molecule with pleiotropic antioxidative properties, would be effective in preventing hepatic I/R injury.

I/R injuries were generated in a model of partial hepatic ischemia in mice. This method produces severe hepatic I/R injuries without mesenteric venous hypertension. Mesenteric congestion is avoided by allowing intestinal blood flow through right and caudate lobes, which leads to a satisfactory survival rate with substantial I/R injuries.

We chose to test mangafodipir for the prevention of I/R injuries because this molecule is known to abrogate ROS-mediated apoptosis/necrosis of hepatocytes in the murine model of acetaminophen-induced acute liver failure [27]. Superoxide anion has been suspected for a long time to be one of the principal actors of liver lesions induced by I/R. Superoxide anion originates from mitochondria or from a cytosolic enzymatic system such as NADPH oxidase or xanthine/xanthine oxidase system. The protective effects of SOD against experimental I/R in transgenic mice overexpressing SOD, have established the role of superoxide anion in I/R injuries of the liver. However, superoxide anion is not the only ROS implicated in I/R. Indeed, high levels of hydrogen peroxide (H_2_O_2_) are produced by Kupffer cells and by parenchymal liver cells following I/R. H_2_O_2_ detoxification by post-ischemic intravenous administration of GSH prevents reperfusion injury in the rat liver after prolonged warm ischemia [22]. Treatment with GSH prevents microcirculatory failure and damages to hepatocytes and improves animal survival. The protection was associated with an increased formation of plasma GSSG, providing evidence of an accelerated detoxification of ROS by intravenously applied GSH [22].

In our model of partial hepatic ischemia, the administration of mangafodipir reduced serum ASAT activities and histological lesions of the liver, diminished the leakage of mitochondrial cytochrome c into the cytosol, and abrogated caspases-3 activation. Altogether, data indicates that mangafodipir ultimately inhibits the apoptosis of hepatocytes that occurs during I/R. Beneficial effects are correlated with the inhibition of ROS production and the preservation of the GSH pool. Mangafodipir, endowed with SOD-like and Glutathione reductase-like activities, allows the detoxification of mitochondrial ROS, and the regeneration of the GSH pool. Thus, the pleiotropic antioxidative properties of mangafodipir can explain its beneficial effects in experimental liver I/R.

The effectiveness of mangafodipir in controlling those parameters was higher than that of IP, which probably acts through other undefined mechanisms. The mild burst of oxidative stress generated during IP activates transcription factors, such as nuclear factor-kappaB and activating protein -1 [11], [34], [35] that might trigger the activity of antioxidant systems [36]–[38]. However, increasing antioxidant systems through IP are very versatile and do not allow the control of the antioxidant status of the liver as well as by a chemical drug.

Mangafodipir has SOD-, catalase-, glutathione reductase-like activities, which contribute to its protective effect against hepatic I/R injury. In a recent study by Llacuna et al [39], I/R of the liver was performed on mice treated with MnTBAP, with BSO or S-adenosylmethionine and demonstrated that SOD mimics present I/R lesion. Moreover, acting on the GSH metabolism by GSH, up regulation by S-adenosylmethionine decrease I/R injuries while GSH depletion by glutathione reductase inhibitor, BSO, potentiate oxidative stress and increase I/R lesions. Since mangafodipir is endowed with both SOD and glutathione reductase-like activity, data indicates that the two enzymatic activities could add an effect to prevent I/R lesions.

In addition to its pharmacological properties, mangafodipir presents pharmacocinetic advantages. Indeed, Mangafodipir trisodium is a tissue-specific imaging agent that concentrates in the liver following *in vivo* administration. Mangafodipir trisodium is a chelate of manganese (II) and of fodipir (DPDP), a vitamin B6 derivate. The fodipir moiety ensures high intra-hepatic concentration of mangafodipir through its specific binding to vitamin B6 receptors borne by hepatocytes. As a contrast agent for liver MRI, mangafodipir has already been safely used in human.

In conclusion, mangafodipir is a major candidate as a molecule used for the prevention of ischemia-reperfusion injury during liver surgery: it exerts a protective effect through its beneficial pharmacological properties and pharmacokinetics. In addition, compared to IC, it reduces blood loss and duration of surgery. Furthermore, mangafodipir is readily available for clinical trials since it has been used for decades as a contrast agent for liver MRI in human.
