# Correction: Mangafodipir Protects against Hepatic Ischemia-Reperfusion Injury in Mice

**DOI:** 10.1371/annotation/cb50dce6-fca7-47b1-b94b-ae21619f26f3

**Published:** 2012-02-01

**Authors:** Romain Coriat, Mahaut Leconte, Niloufar Kavian, Sassia Bedda, Carole Nicco, Christiane Chereau, Claire Goulvestre, Bernard Weill, Alexis Laurent, Frédéric Batteux

There was an error in the Competing Interests declaration. The authors did not declare several potential competing interests relevant to the work reported. They apologize for the omission and would like to disclose the following information: Drs Batteaux and Weill are the founders of Protexel, an R&D company aiming to develop mangafodipir as a commercial drug. Drs Batteaux and Weill are co-inventors of patents describing mangafodipir as a liver-protective and anti-cancer agent. 

